# Omega-3 fatty acids in aromatase inhibitor-associated dyslipidemia: mechanisms, clinical evidence, and future perspectives

**DOI:** 10.3389/fonc.2026.1923962

**Published:** 2026-10-29

**Authors:** Ruiqi Chen, Minghao Xiong, Shengchao Huang

**Affiliations:** Department of Breast Surgery, Affiliated Hospital of Guangdong Medical University, Zhanjiang, China

**Keywords:** AI-associated dyslipidemia, aromatase inhibitors, breast cancer, cardiovascular risk, docosahexaenoic acid, eicosapentaenoic acid, omega-3 fatty acids

## Abstract

Aromatase inhibitors (AIs), including anastrozole, letrozole, and exemestane, are widely used as adjuvant endocrine therapy for postmenopausal women with hormone receptor-positive breast cancer. However, prolonged estrogen deprivation induced by AI therapy may adversely affect lipid homeostasis and has been associated with alterations in low-density lipoprotein cholesterol (LDL-C), total cholesterol (TC), and high-density lipoprotein cholesterol (HDL-C), potentially contributing to long-term cardiovascular risk. The mechanisms underlying these abnormalities appear to involve estrogen-dependent regulation of hepatic lipoprotein metabolism, together with endothelial dysfunction, oxidative stress, and chronic inflammation. Omega-3 polyunsaturated fatty acids, particularly eicosapentaenoic acid (EPA) and docosahexaenoic acid (DHA), have established effects on lipid metabolism and cardiovascular health. Their metabolic actions include activation of peroxisome proliferator-activated receptor-α (PPAR-α), suppression of sterol regulatory element-binding protein-1c (SREBP-1c), modulation of hepatic very-low-density lipoprotein (VLDL) production, enhancement of fatty acid oxidation, and regulation of lipoprotein clearance. However, the effects of EPA and DHA on LDL-C, TC, and HDL-C are less consistent than their triglyceride-lowering effects and may differ according to formulation and dose. In this review, we critically examine the biological mechanisms underlying AI-associated dyslipidemia, compare the lipid effects of individual AIs with tamoxifen, and evaluate the potential mechanisms through which EPA and DHA may modulate cholesterol and lipoprotein metabolism. We further summarize clinical evidence from general dyslipidemia and breast cancer populations and assess its applicability to patients receiving AI therapy. Current evidence supports a strong biological rationale for omega-3 fatty acid supplementation but remains insufficient to establish its efficacy specifically for AI-associated dyslipidemia. Future randomized controlled trials should directly evaluate lipid and cardiovascular outcomes, compare EPA- and DHA-containing formulations, account for differences among individual AIs, and identify patient subgroups most likely to benefit from omega-3-based interventions.

## 1 Introduction

Breast cancer is the most commonly diagnosed cancer among women worldwide, and hormone receptor-positive (HR+) tumors account for the majority of cases (1). or postmenopausal women with HR-positive early-stage breast cancer, third-generation aromatase inhibitors (AIs), including anastrozole, letrozole, and exemestane, are established components of adjuvant endocrine therapy. Randomized controlled trials and patient-level meta-analyses have demonstrated that AIs provide greater protection against breast cancer recurrence than tamoxifen in appropriate patient populations, supporting their widespread use in postmenopausal women with HR-positive disease (2–5).

AIs exert their antitumor effects primarily by inhibiting aromatase-mediated conversion of androgens to estrogens, thereby producing profound estrogen deprivation in postmenopausal women (2, 3). Although sustained suppression of estrogen is central to their therapeutic efficacy, estrogen also contributes to the regulation of lipid and cardiovascular homeostasis. Consequently, prolonged AI therapy may be associated with adverse changes in lipid metabolism, including increases in low-density lipoprotein cholesterol (LDL-C) and total cholesterol (TC), while changes in high-density lipoprotein cholesterol (HDL-C) and triglycerides (TG) appear to be more heterogeneous across studies (3, 6–8). Importantly, a 2024 systematic review and meta-analysis including 15 studies and 1,708 postmenopausal women found that lipid changes varied among individual endocrine therapies, with particularly unfavorable changes observed with letrozole and exemestane, although the magnitude of these changes did not necessarily indicate clinically severe dyslipidemia in patients without pre-existing cardiovascular disease (8).

The potential metabolic consequences of prolonged AI therapy are particularly relevant in breast cancer survivorship. Adjuvant endocrine therapy may be administered for 5 years or longer, with extended treatment considered in selected patients at increased risk of recurrence (3, 5–8). During this period, postmenopausal women may already have an unfavorable metabolic profile related to age and physiological estrogen decline, potentially increasing susceptibility to treatment-associated lipid abnormalities and cardiovascular risk (1, 2). Therefore, assessment and management of cardiovascular and metabolic risk factors represent important components of long-term care for breast cancer survivors receiving AI therapy (6–8). However, the magnitude and clinical significance of AI-associated lipid alterations are not uniform, and differences among individual AIs and comparisons with tamoxifen suggest that the relationship between estrogen deprivation and lipid metabolism is more complex than a simple increase in cardiovascular risk.

Omega-3 polyunsaturated fatty acids (n-3 PUFAs), particularly eicosapentaenoic acid (EPA) and docosahexaenoic acid (DHA), have attracted interest as potential adjunctive interventions for metabolic abnormalities because of their established effects on triglyceride-rich lipoprotein metabolism and their broader biological effects on inflammation and vascular function.Clinical studies have consistently demonstrated triglyceride-lowering effects of pharmacological doses of EPA and/or DHA, whereas their effects on LDL-C, TC, and HDL-C are generally smaller and less consistent (9, 10). Prescription omega-3 preparations have therefore been investigated primarily in patients with hypertriglyceridemia, and their clinical use should be distinguished from the effects of dietary omega-3 intake or non-prescription supplements (11). In addition, epidemiological and clinical studies have suggested potential associations between omega-3 exposure and breast cancer-related outcomes, although these findings do not establish a therapeutic role for omega-3 fatty acids in patients receiving endocrine therapy (10, 12).

Despite this biological rationale, the evidence specifically addressing omega-3 fatty acids in AI-associated dyslipidemia remains limited. Much of the available evidence is derived from patients with hypertriglyceridemia, established cardiovascular disease, or broader breast cancer populations rather than from patients receiving long-term AI therapy (9–12). Moreover, EPA and DHA are not metabolically interchangeable, and their effects may vary according to formulation, dose, baseline lipid phenotype, and clinical context. These considerations are particularly important because evidence of cardiovascular benefit obtained with specific EPA formulations in selected high-risk populations cannot necessarily be extrapolated to combined EPA/DHA preparations or to breast cancer survivors receiving AIs.

Accordingly, this narrative review aims to critically synthesize the current evidence on AI-associated dyslipidemia and the potential role of omega-3 fatty acids in its management. We first examine the clinical characteristics and biological mechanisms linking AI-induced estrogen deprivation with alterations in LDL-C, TC, HDL-C, and TG. We then compare the lipid and metabolic effects of anastrozole, letrozole, and exemestane with those of tamoxifen, with particular attention to potential mechanistic differences among individual endocrine therapies. Finally, we evaluate the mechanisms by which EPA and DHA may influence triglyceride-rich lipoprotein and cholesterol metabolism, critically assess clinical evidence from non-breast cancer and breast cancer populations, and identify the key evidence gaps that must be addressed before omega-3 fatty acids can be considered an established therapeutic strategy for AI-associated dyslipidemia.

## 2 Review methodology

### 2.1 Literature search strategy

This narrative review was developed through targeted searches of PubMed/MEDLINE, Embase, and the Cochrane Library using combinations of terms related to aromatase inhibitors, anastrozole, letrozole, exemestane, tamoxifen, dyslipidemia, LDL-C, HDL-C, total cholesterol, triglycerides, omega-3 fatty acids, EPA, and DHA. Additional relevant studies were identified through reference-list screening of major reviews, meta-analyses, and clinical trials.

### 2.2 Evidence selection and synthesis

Priority was given to randomized controlled trials, systematic reviews and meta-analyses, prospective clinical studies, and mechanistic investigations that directly addressed AI-associated lipid abnormalities, omega-3-mediated lipid metabolism, cardiovascular outcomes, or breast cancer survivorship. Studies were selected for their clinical relevance, methodological importance, sample size, or contribution to understanding lipid or cardiovascular outcomes. Because this article is a narrative review rather than a systematic review, no formal quantitative meta-analysis was performed. Evidence was synthesized according to the strength and directness of evidence, with particular distinction between studies conducted in AI-treated breast cancer populations and those conducted in broader dyslipidemia or cardiovascular populations.

## 3 Aromatase inhibitor-associated metabolic abnormalities: disease background and clinical implications

### 3.1 Clinical applications and global trends in the use of AIs

Third-generation aromatase inhibitors (AIs) have become a standard component of adjuvant endocrine therapy for postmenopausal women with hormone receptor-positive (HR+) breast cancer. A longitudinal international drug utilization study conducted across nine developed countries between 2001 and 2012 demonstrated a consistent increase in AI use over time. By 2012, AIs accounted for more than half of all endocrine therapy prescriptions in each participating country, with utilization rates reaching 74% in France and 80% in Denmark (13).

Over the past decade, the clinical indications for AIs have expanded beyond the conventional postmenopausal population to include selected premenopausal women receiving ovarian function suppression. In parallel, accumulating evidence has supported the consideration of extended endocrine therapy, with treatment durations of up to 10 years being considered for selected patients, rather than the traditional 5-year course. Consequently, cumulative exposure to AIs among breast cancer survivors has increased (3, 5). The prolonged duration of endocrine therapy has further underscored the importance of monitoring and managing treatment-related adverse effects, particularly metabolic and cardiovascular complications, as integral components of long-term survivorship care and clinical decision-making (3, 5).

### 3.2 Prevalence and characteristics of hyperlipidaemia associated with AIs

With the widespread use of aromatase inhibitors (AIs) in the adjuvant endocrine treatment of breast cancer, dyslipidemia has emerged as one of the most frequently reported metabolic adverse effects. The reported incidence of AI-associated dyslipidemia varies across studies, potentially reflecting differences in patient characteristics, baseline metabolic status, AI subtype, follow-up duration, and study design. Nevertheless, accumulating evidence suggests that long-term AI therapy may be associated with adverse alterations in lipid metabolism. The major clinical findings are summarized in **Table 1** (6–8).

**Table 1:** Major clinical studies evaluating the effects of aromatase inhibitors on lipid metabolism and cardiovascular outcomes.

Study	Design	Sample size	AI type	Main findings
BIG 1–98 Trial (4)	RCT	8,010	Letrozole	Increased incidence of hypercholesterolemia compared with tamoxifen
IES Trial (5)	RCT	4,724	Exemestane	Exemestane showed relatively limited changes in lipid parameters compared with some non-steroidal AIs
EBCTCG 2015 (3)	Patient-level meta-analysis	31,920	Multiple AIs	AIs reduced breast cancer recurrence and mortality compared with tamoxifen; non-breast-cancer mortality was similar
Amir et al., 2011 (6)	Systematic review and meta-analysis	30,023; 7 RCTs	Multiple AIs	AI use was associated with higher odds of cardiovascular disease (OR 1.26, 95% CI 1.10–1.43)
Yoo et al., 2023 (7)	Meta-analysis	25 studies; outcome-specific N	Multiple AIs	HDL-C decreased significantly at 6 months; LDL-C was higher versus tamoxifen at 6 months, whereas changes versus baseline were heterogeneous
Bérczi et al., 2024 (8)	Meta-analysis	1,708; 15 studies	Multiple AIs	Lipid responses varied by AI and treatment duration; letrozole and exemestane showed relatively less favorable patterns

AI, aromatase inhibitor; RCT, randomized controlled trial; LDL-C, low-density lipoprotein cholesterol; HDL-C, high-density lipoprotein cholesterol; TC, total cholesterol; TG, triglycerides.

Several systematic reviews and meta-analyses have indicated that AIs are generally associated with increases in low-density lipoprotein cholesterol (LDL-C) and total cholesterol (TC) levels, whereas reductions in high-density lipoprotein cholesterol (HDL-C) have also been reported. In contrast, the effects of AIs on triglyceride (TG) levels remain inconsistent across studies (6–8). Notably, the recent meta-analysis by Bérczi et al. further demonstrated significant alterations in the lipid profile of postmenopausal women treated with AIs, reinforcing concerns regarding the potential long-term metabolic consequences of estrogen deprivation (8).

Beyond the lipid abnormalities themselves, AI-associated dyslipidemia may contribute to an increased risk of atherosclerosis and ischemic cardiovascular events, particularly among patients receiving long-term endocrine therapy (6, 7). As the duration of adjuvant endocrine therapy has been extended from 5 to 10 years in selected high-risk populations, the cumulative metabolic burden associated with prolonged AI exposure has become an increasingly relevant clinical concern (3, 6, 7). Therefore, early identification and appropriate management of dyslipidemia may be important components of survivorship care to help preserve long-term cardiovascular health and quality of life among breast cancer survivors (**Table 1**).

### 3.3 Differential effects of different AI types on lipid profiles

Although all aromatase inhibitors (AIs) suppress estrogen synthesis by inhibiting aromatase activity, their effects on lipid metabolism may differ because of differences in their chemical structures and pharmacological properties. Currently used third-generation AIs can be broadly classified into two groups: the non-steroidal aromatase inhibitors anastrozole and letrozole, and the steroidal aromatase inactivator exemestane, which exerts its effects through a distinct pharmacological mechanism (2).

Tamoxifen, although not an AI, is an important clinical comparator when interpreting the metabolic effects of AIs. Unlike AIs, tamoxifen is a selective estrogen receptor modulator (SERM) and may exert relatively favorable effects on total cholesterol and LDL-C. A systematic review and meta-analysis of 18 randomized controlled trials found that tamoxifen significantly reduced TC and LDL-C compared with placebo, although its effects on HDL-C and TG were less consistent (14). Therefore, differences in lipid profiles between AIs and tamoxifen may reflect not only estrogen deprivation induced by aromatase inhibition but also the intrinsic lipid-modifying effects of tamoxifen.

Randomized clinical trials have demonstrated differences in lipid-related outcomes among individual AIs. In the BIG 1–98 trial, hypercholesterolemia was reported more frequently in patients receiving letrozole than in those receiving tamoxifen, indicating a potential association between letrozole treatment and less favorable lipid profiles (4). These findings indicate that the less favorable lipid profile observed with letrozole relative to tamoxifen may reflect both estrogen deprivation induced by AI therapy and the lipid-modifying effects of tamoxifen.

The ATAC trial similarly reported dyslipidemia and hypercholesterolemia among patients receiving anastrozole. However, the reported magnitude of lipid abnormalities appeared to be relatively modest compared with the changes reported in studies of letrozole. It similarly reported a higher incidence of hypercholesterolemia with anastrozole than with tamoxifen, although the magnitude and consistency of changes in individual lipid parameters have varied across studies (15). Importantly, the occurrence of hypercholesterolemia should not be interpreted as evidence that anastrozole uniformly increases all atherogenic lipid parameters. Rather, subsequent evidence suggests that the effects of anastrozole on individual lipid measures are heterogeneous.

The Intergroup Exemestane Study (IES) and subsequent metabolic analyses suggested a comparatively limited effect of exemestane on TC and LDL-C. Some studies have also reported reductions in TG concentrations, although modest decreases in HDL-C have been described (5, 16). These findings suggest that exemestane may have a comparatively attenuated or heterogeneous effect on conventional lipid parameters compared with some non-steroidal AIs.

Recent systematic evidence further highlights the heterogeneity in lipid profiles associated with individual AIs (8). In a 2024 systematic review and meta-analysis of 15 studies involving 1,708 postmenopausal women with breast cancer, lipid responses differed according to AI type and treatment duration. Letrozole was associated with an increase in TC at 3 months, whereas exemestane was associated with a reduction in TC at the same time point. Changes in LDL-C, HDL-C, and TG also varied according to AI type and treatment duration. These findings indicate that the lipid effects of AIs should be interpreted according to the specific agent and treatment duration rather than considered a uniform class effect (8). The relatively distinct lipid profile observed with exemestane may partly reflect its steroidal structure and weak androgenic properties, which could influence hepatic lipoprotein metabolism through mechanisms that differ from those of non-steroidal AIs (16).

Importantly, previous tamoxifen exposure may influence the interpretation of lipid changes observed after initiation of AI therapy. In a study of postmenopausal women with early-stage breast cancer treated with exemestane or letrozole, LDL-C increased significantly in the overall cohort, but this increase was observed predominantly among patients with prior tamoxifen exposure. Prior tamoxifen use was also associated with a greater increase in LDL-C, suggesting that withdrawal of tamoxifen-associated lipid-modifying effects may contribute, at least in part, to the lipid changes observed after switching to an AI (17). However, this finding should be interpreted cautiously because the study was not designed to establish a causal effect of tamoxifen withdrawal, and the influence of prior endocrine therapy may be confounded by differences in treatment sequence and baseline metabolic characteristics.

This interpretation is broadly consistent with more recent pooled evidence showing substantial heterogeneity in lipid responses among individual endocrine therapies. A 2024 systematic review and meta-analysis of 15 studies involving 1,708 patients found that lipid responses varied according to AI type and treatment duration. Letrozole was associated with an increase in TC and LDL-C at 3 months, whereas changes with anastrozole were less pronounced and exemestane showed a different pattern of lipid responses. Tamoxifen was associated with reductions in TC and LDL-C in the available subgroups, further supporting its role as an important pharmacological comparator rather than an AI subtype (8). However, the heterogeneity among treatment regimens and the limited number of studies available for some comparisons preclude definitive conclusions regarding the superiority of one endocrine therapy over another with respect to long-term lipid outcomes (8).

A comparison of lipid profile effects across individual AIs is summarized in **Table 2**.

**Table 2:** Comparative effects of individual aromatase inhibitors on lipid profiles.

Parameter	Anastrozole	Letrozole	Exemestane
Drug class	Non-steroidal AI	Non-steroidal AI	Steroidal AI
Mechanism	Reversible aromatase inhibition	Reversible aromatase inhibition	Irreversible aromatase inactivation
LDL-C	↔ / variable	↑ / variable	↔ / variable
TC	↔ / variable	↑ / variable	↔ / variable
HDL-C	↔ / variable	↔ / ↓	↓ / variable
TG	↔ / variable	↔ / ↑	↓ / ↔
Overall lipid effect	Relatively heterogeneous	Relatively less favorable in some studies	Relatively attenuated / heterogeneous
Key supporting studies	ATAC (15)	BIG 1-98 (4)	IES (5); TEAM Greek Sub-study (16)

AI, aromatase inhibitor; LDL-C, low-density lipoprotein cholesterol; HDL-C, high-density lipoprotein cholesterol; TG, triglycerides; Note: ↔, no consistent significant change; ↑, increase; ↓, decrease. The direction of lipid changes represents the overall pattern reported across clinical studies and does not imply a uniform effect in all patients. Lipid responses may vary according to baseline lipid profile, treatment duration, previous endocrine therapy, concomitant medications, and study design. Tamoxifen is not included as an AI subtype because it is a selective estrogen receptor modulator rather than an aromatase inhibitor; it is discussed in the main text as a clinically relevant comparator.

### 3.4 Potential mechanisms of AIs-associated hyperlipidaemia and cardiovascular risk

Aromatase inhibitors (AIs) are established endocrine therapies for patients with hormone receptor-positive breast cancer. However, prolonged estrogen suppression during AI therapy may adversely affect lipid metabolism and cardiovascular homeostasis. AI-associated dyslipidemia is likely to result from multiple interacting mechanisms rather than a single metabolic pathway. These mechanisms include estrogen deficiency, alterations in lipid metabolism, endothelial dysfunction, and chronic low-grade inflammation, which may collectively contribute to metabolic and cardiovascular abnormalities (1, 6–8).

Estrogen plays an important role in maintaining lipid homeostasis by regulating hepatic lipoprotein metabolism, LDL-C clearance, and HDL-C metabolism (1). AIs inhibit aromatase activity and thereby reduce the conversion of androgens to estrogens. In postmenopausal women, this results in sustained estrogen deprivation (2, 3). Reduced estrogen signaling may alter hepatic lipid metabolism and lipoprotein turnover, contributing particularly to increases in LDL-C and TC, whereas changes in HDL-C appear to be more variable (3, 6, 8).

The resulting adverse lipid profile may contribute to atherosclerotic cardiovascular risk through several complementary pathways. Elevated LDL-C and TC may promote atherogenic lipoprotein accumulation, while estrogen deficiency may additionally affect endothelial function and vascular homeostasis. Oxidative stress and chronic low-grade inflammation may further contribute to endothelial dysfunction and atherosclerotic progression (6, 7). However, the extent to which AI-associated lipid changes independently account for cardiovascular events remains uncertain, as cardiovascular risk in breast cancer survivors is influenced by multiple factors, including age, baseline cardiovascular risk, menopausal status, comorbidities, and concomitant therapies.

Taken together, the metabolic effects of AI therapy appear to be multifactorial rather than attributable solely to changes in circulating lipid concentrations. This provides a biological rationale for continued monitoring of lipid profiles and cardiovascular risk during long-term AI therapy and supports further investigation of potential adjunctive interventions targeting AI-associated metabolic abnormalities.

**Figure 1** summarizes the proposed pathways linking AI therapy with dyslipidemia and cardiovascular risk based on the mechanisms supported by current evidence.

**Figure 1:**
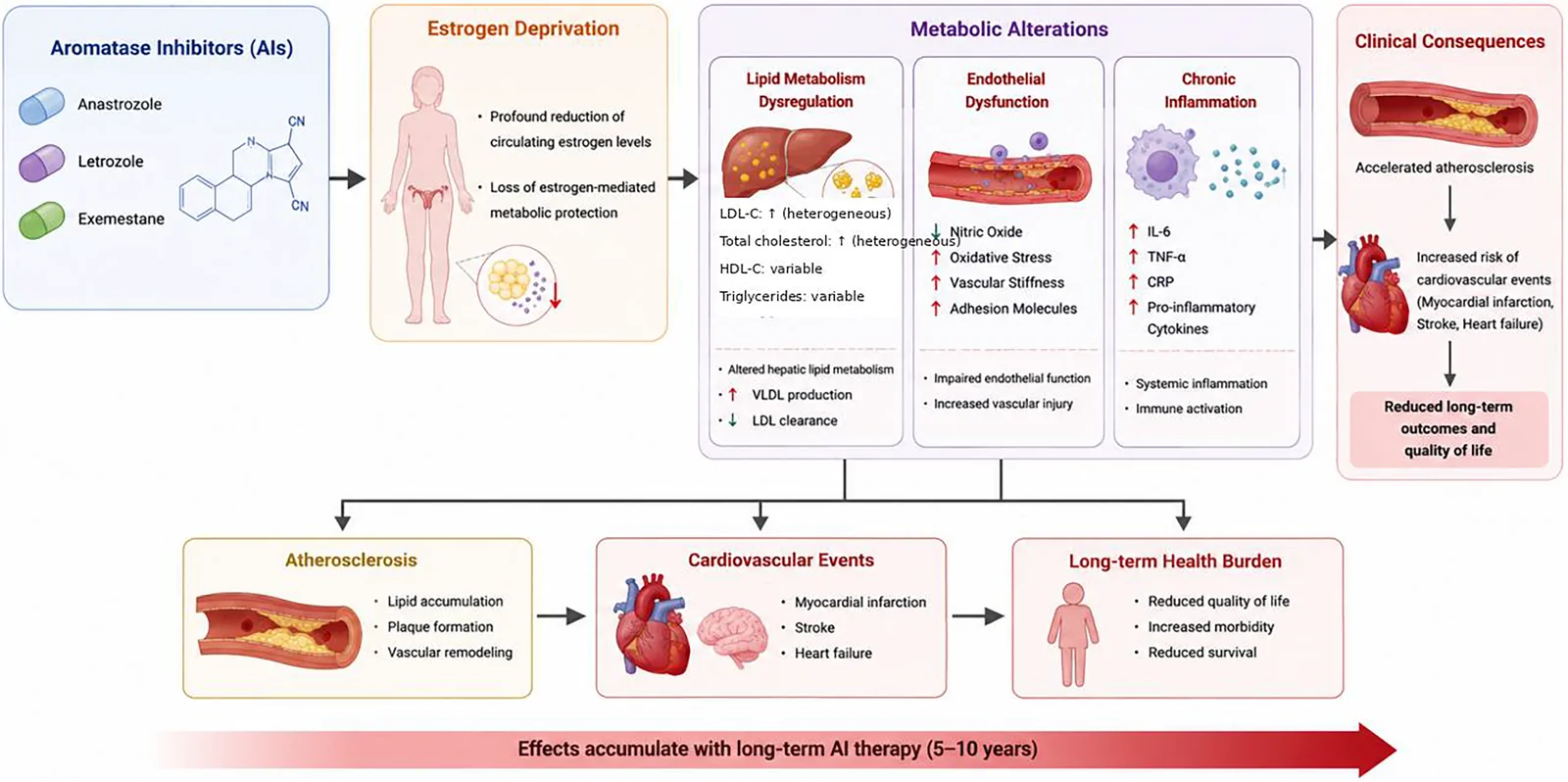
Pathophysiological mechanisms underlying aromatase inhibitor–associated dyslipidemia and cardiovascular risk. Aromatase inhibitor (AI) therapy suppresses estrogen synthesis, resulting in persistent estrogen deprivation. Reduced estrogen levels may alter hepatic lipoprotein metabolism, with increases in low-density lipoprotein cholesterol (LDL-C) and total cholesterol (TC) reported in some studies, whereas changes in high-density lipoprotein cholesterol (HDL-C) and triglycerides are more heterogeneous. Estrogen deficiency also impairs endothelial function through reduced nitric oxide bioavailability and enhanced oxidative stress, while simultaneously promoting chronic low-grade inflammation characterized by elevated interleukin-6 (IL-6), tumor necrosis factor-α (TNF-α), and C-reactive protein (CRP). These interconnected mechanisms contribute to atherosclerosis, increase cardiovascular risk, and may ultimately impair long-term health outcomes in breast cancer survivors receiving prolonged AI therapy. AI, aromatase inhibitor; LDL-C, low-density lipoprotein cholesterol; HDL-C, high-density lipoprotein cholesterol; VLDL, very-low-density lipoprotein; IL-6, interleukin-6; TNF-α, tumor necrosis factor-α; CRP, C-reactive protein.

## 4 Omega-3 fatty acids: biological mechanisms and metabolic effects

### 4.1 Biological Rationale for EPA and DHA

Omega-3 polyunsaturated fatty acids (n-3 PUFAs), particularly eicosapentaenoic acid (EPA) and docosahexaenoic acid (DHA), are long-chain fatty acids that exert metabolic effects through both structural and transcriptional mechanisms. Following dietary intake, EPA and DHA are incorporated into membrane phospholipids and circulating lipoproteins, thereby modifying membrane fatty acid composition, cellular signaling, and lipid metabolism (9, 18). Their biological effects are mediated not only by their incorporation into cellular membranes but also by regulation of nuclear transcription factors involved in fatty acid synthesis and oxidation, including peroxisome proliferator-activated receptors (PPARs) and sterol regulatory element-binding protein-1c (SREBP-1c) (19, 20).

At the hepatic level, n-3 PUFAs shift lipid metabolism away from *de novo* lipogenesis and toward fatty acid oxidation. Activation of PPAR-dependent pathways promotes fatty acid β-oxidation, whereas suppression of SREBP-1c reduces the transcription of genes involved in fatty acid and triglyceride synthesis (19–21). These coordinated effects decrease the availability of fatty acids for hepatic triglyceride synthesis and consequently reduce the substrate available for very-low-density lipoprotein (VLDL) production (21, 22). Experimental and clinical evidence therefore supports a role for EPA and DHA in regulating hepatic lipid flux rather than simply acting as passive dietary fatty acids (19, 21, 22). These complementary mechanisms are summarized in **Figure 2**.

**Figure 2:**
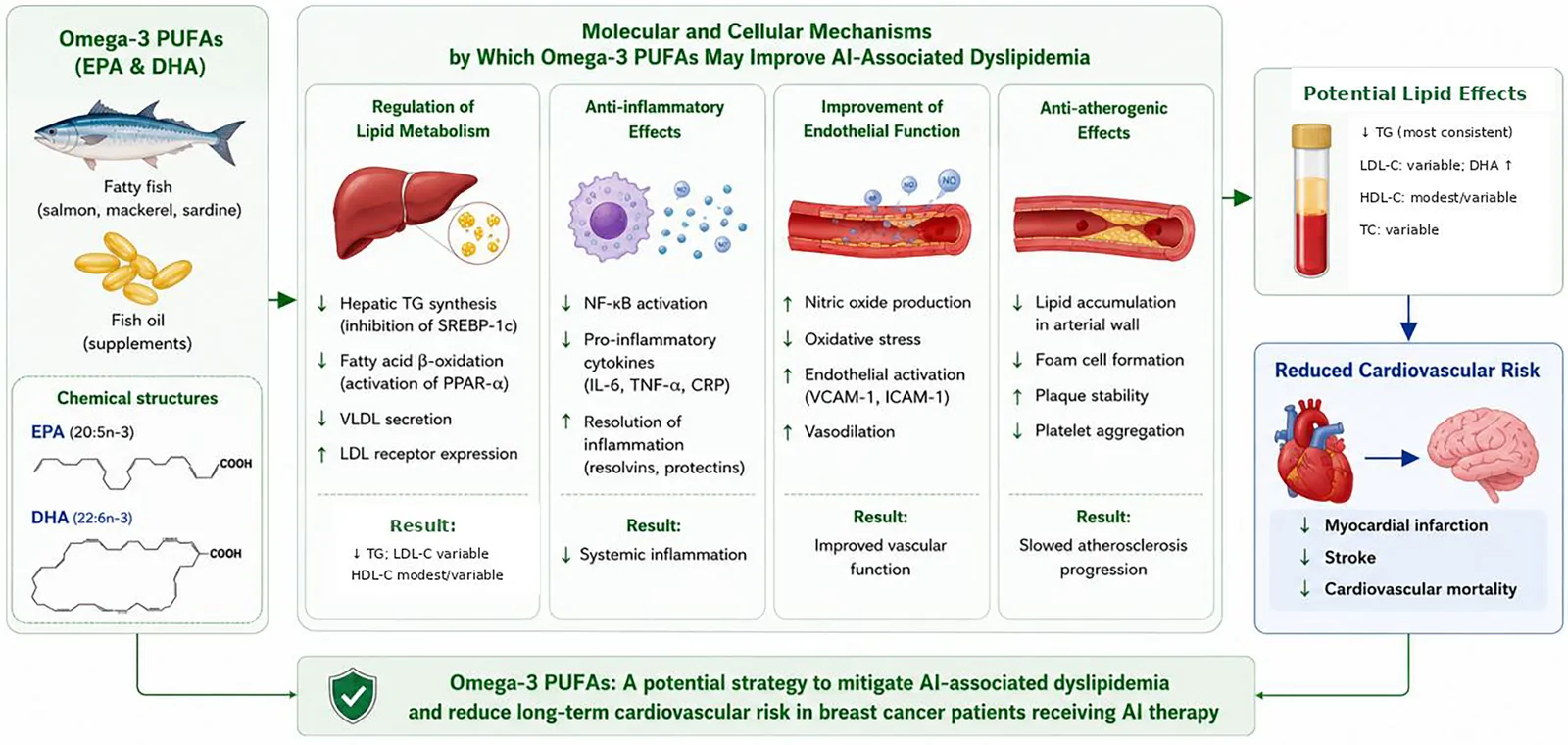
Proposed mechanisms by which omega-3 fatty acids may mitigate AI-associated dyslipidemia and cardiovascular risk. Omega-3 polyunsaturated fatty acids (PUFAs), particularly eicosapentaenoic acid (EPA) and docosahexaenoic acid (DHA), may improve AI-associated dyslipidemia through multiple complementary mechanisms. These include regulation of hepatic lipid metabolism via activation of PPAR-α and suppression of SREBP-1c signaling, attenuation of chronic inflammation through inhibition of NF-κB–mediated cytokine production, improvement of endothelial function by enhancing nitric oxide bioavailability and reducing endothelial activation, and inhibition of atherosclerotic progression. Collectively, these effects contribute to improved lipid profiles and reduced cardiovascular risk in patients with breast cancer receiving long-term aromatase inhibitor therapy. PUFAs, polyunsaturated fatty acids; EPA, eicosapentaenoic acid; DHA, docosahexaenoic acid; TG, triglyceride; LDL-C, low-density lipoprotein cholesterol; HDL-C, high-density lipoprotein cholesterol; SREBP-1c, sterol regulatory element-binding protein-1c; PPAR-α, peroxisome proliferator-activated receptor-α; NF-κB, nuclear factor kappa B; IL-6, interleukin-6; TNF-α, tumor necrosis factor-α; CRP, C-reactive protein; VCAM-1, vascular cell adhesion molecule-1; ICAM-1, intercellular adhesion molecule-1; NO, nitric oxide.

EPA and DHA, however, are metabolically related but not interchangeable. DHA can undergo retroconversion to EPA, whereas conversion of EPA to DHA appears to be negligible in humans. In addition, DHA is preferentially incorporated into triglycerides, whereas EPA is more evenly distributed among triglycerides, cholesterol esters, and phospholipids (22). These differences in tissue and lipoprotein incorporation may contribute to their distinct effects on circulating lipoproteins and provide a biological basis for considering EPA- and DHA-containing preparations separately in clinical studies.

### 4.2 Effects on triglyceride-rich lipoprotein metabolism

The most consistent lipid effect of marine-derived omega-3 fatty acids is a reduction in circulating triglyceride (TG) concentrations (21–26). This effect is primarily mediated through coordinated changes in hepatic TG synthesis, VLDL production, and peripheral clearance of TG-rich lipoproteins rather than through a single metabolic pathway (21, 22).

At the hepatic level, EPA and DHA suppress *de novo* lipogenesis through downregulation of SREBP-1c and promote fatty acid oxidation through activation of PPAR-dependent pathways (20–22). Increased β-oxidation decreases the availability of fatty acid substrates for TG synthesis, while inhibition of enzymes involved in hepatic TG synthesis further limits the incorporation of fatty acids into TG molecules. These effects collectively reduce hepatic TG availability and VLDL-TG production.Human mechanistic studies indicate that reduced hepatic VLDL production is a major determinant of the fasting TG-lowering effect of EPA and DHA (21, 22).

Omega-3 fatty acids also influence the clearance of circulating TG-rich lipoproteins. EPA and DHA increase postprandial lipoprotein lipase (LPL)-mediated hydrolysis of TG within VLDL and chylomicrons, thereby facilitating the clearance of TG-rich particles from the circulation (21, 22). Reduction in VLDL-TG concentrations and enhanced chylomicron clearance therefore contribute to the reduction in postprandial TG levels. Importantly, available human evidence does not demonstrate a clear difference between EPA and DHA in their overall ability to reduce fasting or postprandial TG concentrations (22).

Importantly, the TG-lowering effect should not be interpreted as evidence that omega-3 fatty acids uniformly improve all lipid fractions. The metabolic consequences of reducing VLDL-TG production can alter downstream lipoprotein remodeling, resulting in different effects on LDL-C and HDL-C (22, 27). Therefore, evaluation of omega-3 therapy should consider changes across the lipid profile rather than focusing exclusively on TG.

### 4.3 Effects on LDL-C, TC, and HDL-C

In contrast to the relatively consistent reduction in TG, the effects of omega-3 fatty acids on total cholesterol (TC), LDL-C, and HDL-C are smaller and more heterogeneous (23, 24, 27, 28). This distinction is clinically important because AI-associated dyslipidemia may involve changes in multiple lipid fractions rather than isolated hypertriglyceridemia (7, 8). Accordingly, the potential role of omega-3 fatty acids in AI-associated metabolic abnormalities cannot be assessed solely on the basis of their TG-lowering capacity.

The effect on LDL-C appears to depend substantially on the composition of the omega-3 preparation. A systematic review and meta-analysis directly comparing EPA and DHA found that both fatty acids reduced TG concentrations, but DHA increased LDL-C whereas EPA had a comparatively neutral effect (27). In direct comparison studies, DHA increased LDL-C more than EPA, while DHA also produced a greater increase in HDL-C. Thus, the commonly observed increase in LDL-C with some omega-3 preparations should not necessarily be attributed to omega-3 fatty acids as a homogeneous class. Rather, it appears to be influenced by the relative contribution of DHA and EPA (27).

Several mechanisms may explain the differential effects on LDL-C. Reduction in hepatic VLDL-TG production accelerates the remodeling of VLDL particles and their conversion through intermediate-density lipoprotein (IDL) toward LDL particles. Consequently, although the concentration of TG-rich VLDL particles decreases, the flux of lipoprotein particles through the VLDL–IDL–LDL pathway may increase, potentially contributing to a modest elevation in LDL-C with DHA-containing preparations. The overall LDL response is also influenced by lipoprotein metabolism and interindividual factors, including genetic variation such as APOE genotype (22).

The effects on HDL-C are generally modest. Evidence from direct comparisons indicates that DHA can increase HDL-C, whereas EPA has a smaller or nonsignificant effect (27). This difference further supports the concept that EPA and DHA should not be regarded as metabolically equivalent interventions. Importantly, an increase in HDL-C should not automatically be interpreted as cardiovascular benefit, because changes in HDL-C concentration do not necessarily reflect improvements in HDL functionality or cardiovascular outcomes (24, 27).

Changes in TC are generally less pronounced and less consistent than changes in TG (24, 28). Because TC reflects the combined contribution of multiple lipoprotein fractions, the net effect of omega-3 supplementation depends on the balance between reductions in VLDL-associated TG, changes in LDL-C, and changes in HDL-C (22, 27). Consequently, omega-3 fatty acids should primarily be regarded as TG-lowering agents rather than broad-spectrum cholesterol-lowering therapies (24, 25, 28).

This distinction is particularly relevant when considering AI-associated dyslipidemia. If AI therapy predominantly alters LDL-C or TC rather than TG, the lipid-modifying effects observed with high-dose omega-3 therapy in conventional hypertriglyceridemia cannot be directly extrapolated to patients receiving AIs. Conversely, patients with AI-associated hypertriglyceridemia may represent a biologically plausible subgroup in whom the established TG-lowering effects of EPA and DHA are more directly relevant.

### 4.4 EPA- and DHA-specific mechanisms

Although EPA and DHA share several metabolic pathways, accumulating evidence indicates that they should be considered as distinct bioactive fatty acids rather than interchangeable components of omega-3 therapy (22, 27, 29). Both fatty acids reduce circulating TG primarily through decreased hepatic VLDL-TG production and enhanced postprandial clearance of TG-rich lipoproteins. However, their effects on cholesterol-containing lipoproteins differ more consistently than their effects on TG (22, 27).

EPA appears to have a relatively neutral effect on LDL-C, whereas DHA is more consistently associated with an increase in LDL-C. In a systematic review and meta-analysis of randomized trials, DHA increased LDL-C compared with placebo, while EPA did not significantly increase LDL-C; in direct EPA-versus-DHA comparisons, DHA produced a significantly greater increase in LDL-C than EPA. DHA also increased HDL-C, whereas the effect of EPA on HDL-C was comparatively limited. These findings indicate that the lipid effects of omega-3 therapy depend not only on dose but also on the specific fatty acid administered (27).

The differential effects of EPA and DHA may partly reflect differences in their intracellular and lipoprotein metabolism. These differences have important implications for interpreting clinical evidence. Cardiovascular outcome benefits observed with purified EPA therapy, particularly icosapent ethyl, cannot automatically be extrapolated to combined EPA+DHA preparations (18, 30–32). The REDUCE-IT trial demonstrated a substantial reduction in cardiovascular events with high-dose icosapent ethyl in selected statin-treated high-risk patients (31), while evidence from broader omega-3 preparations has been more heterogeneous (28, 32, 33). Thus, future studies investigating omega-3 supplementation in AI-treated patients with breast cancer should specify the EPA: DHA composition, formulation, and dose rather than grouping all omega-3 preparations into a single intervention category.

## 5 Clinical evidence of omega-3 fatty acids

### 5.1 Evidence for lipid modulation by omega-3 fatty acids in non-breast cancer populations

Randomized controlled trials and meta-analyses have consistently demonstrated that the principal lipid-lowering effect of marine-derived omega-3 fatty acids is a reduction in triglyceride (TG) concentrations, with the magnitude of response influenced by baseline TG levels, dose, and formulation (25, 26). However, effects on total cholesterol (TC), LDL-C, and HDL-C are generally smaller and less consistent than those observed for TG.

The MARINE trial evaluated icosapent ethyl, a highly purified EPA formulation, in patients with severe hypertriglyceridemia (TG ≥500 mg/dL). Treatment with 4 g/day reduced TG concentrations by approximately 33% over 12 weeks without a clinically important increase in LDL-C (34). The ANCHOR trial subsequently demonstrated reductions in TG, non-HDL-C, and apolipoprotein B among statin-treated patients with persistent hypertriglyceridemia (35). These findings provide strong evidence for the triglyceride-lowering efficacy of prescription-grade omega-3 formulations, particularly high-dose EPA, in patients with hypertriglyceridemia.

Evidence from systematic reviews further supports a dose-response relationship. A Cochrane systematic review found that omega-3 supplementation reduced TG concentrations, whereas effects on TC and LDL-C were relatively modest (28). A dose-response meta-analysis also suggested that greater EPA/DHA intake was associated with greater TG reduction, with clinically relevant effects generally observed at approximately 2–4 g/day (25). Importantly, the lipid effects of EPA and DHA are not identical. Evidence suggests that DHA may increase LDL-C to a greater extent than EPA, whereas EPA has a comparatively neutral effect on LDL-C (27).

The cardiovascular effects of omega-3 fatty acids also appear to depend strongly on formulation, dose, and baseline cardiovascular risk. In REDUCE-IT, icosapent ethyl at 4 g/day reduced major adverse cardiovascular events by 25% among statin-treated patients with elevated TG and established cardiovascular disease or diabetes plus additional risk factors (31). Similarly, JELIS reported a reduction in major coronary events with EPA supplementation added to statin therapy (18). However, these findings should not be generalized to all omega-3 formulations or to populations without comparable cardiovascular risk.

Taken together, the strongest clinical evidence supports high-dose prescription omega-3 therapy primarily as a triglyceride-lowering intervention, particularly in patients with hypertriglyceridemia. Evidence for substantial LDL-C reduction is limited, and cardiovascular outcome benefits appear to be formulation- and population-specific. Therefore, these findings provide biological and clinical rationale for investigating omega-3 fatty acids in AI-associated dyslipidemia but cannot, by themselves, establish efficacy in patients with breast cancer receiving AIs.

### 5.2 Omega-3 fatty acids and metabolic health in patients with breast cancer

Evidence directly evaluating omega-3 fatty acid supplementation in breast cancer populations remains limited compared with the extensive literature in patients with conventional dyslipidemia or cardiovascular disease. Available studies have primarily examined omega-3 incorporation into breast tissue, inflammatory biomarkers, nutritional status, treatment-related symptoms, or broader survivorship outcomes, rather than AI-associated lipid abnormalities as primary endpoints.

In women at high risk of breast cancer, Yee et al. demonstrated dose-dependent incorporation of EPA and DHA into breast adipose tissue following supplementation (12). This finding indicates that orally administered omega-3 fatty acids can achieve measurable tissue exposure in breast cancer-related populations and provides biological support for their potential metabolic effects.

A meta-analysis by Yang et al. reported an association between higher exposure to long-chain n-3 polyunsaturated fatty acids and more favorable breast cancer-related outcomes; however, the observational nature of the underlying evidence limits causal inference (10). Similarly, reviews examining omega-3 fatty acids in breast cancer prevention and survivorship have suggested potential benefits related to inflammation, metabolic health, and treatment-related outcomes, while emphasizing the need for prospective clinical trials (36). A more recent systematic review likewise reported potential improvements in metabolic status, inflammatory markers, and patient-reported outcomes among women with breast cancer receiving omega-3 interventions (37).

However, these findings should not be interpreted as direct evidence that omega-3 fatty acids prevent or treat AI-associated dyslipidemia. Most studies were conducted in heterogeneous breast cancer populations and generally did not stratify participants according to endocrine therapy, AI subtype, baseline lipid profile, or cardiovascular risk. Moreover, lipid parameters were rarely prespecified as primary endpoints. Consequently, the current evidence supports the biological feasibility and potential metabolic relevance of omega-3 supplementation but remains insufficient to determine its clinical efficacy for dyslipidemia specifically associated with AI therapy.

### 5.3 Evidence and limitations in AI-associated dyslipidemia

The evidence supporting omega-3 fatty acids specifically for AI-associated dyslipidemia remains limited. To date, no large randomized controlled trial has directly evaluated the efficacy of omega-3 supplementation for dyslipidemia in patients with breast cancer receiving long-term AI therapy. Consequently, the current evidence must be interpreted as an indirect chain of evidence comprising mechanistic studies, clinical trials in patients with conventional hypertriglyceridemia, and studies of omega-3 supplementation in broader breast cancer populations.

From a mechanistic perspective, AI-induced estrogen deprivation may contribute to alterations in hepatic lipid metabolism, endothelial dysfunction, chronic inflammation, and oxidative stress. Omega-3 fatty acids may theoretically counteract some of these abnormalities by enhancing hepatic fatty acid oxidation, reducing VLDL production, modulating lipoprotein metabolism, and attenuating inflammatory signaling (9, 25). EPA and DHA may therefore have complementary effects that could be relevant to the metabolic phenotype associated with prolonged AI therapy (**Figure 3**).

**Figure 3:**
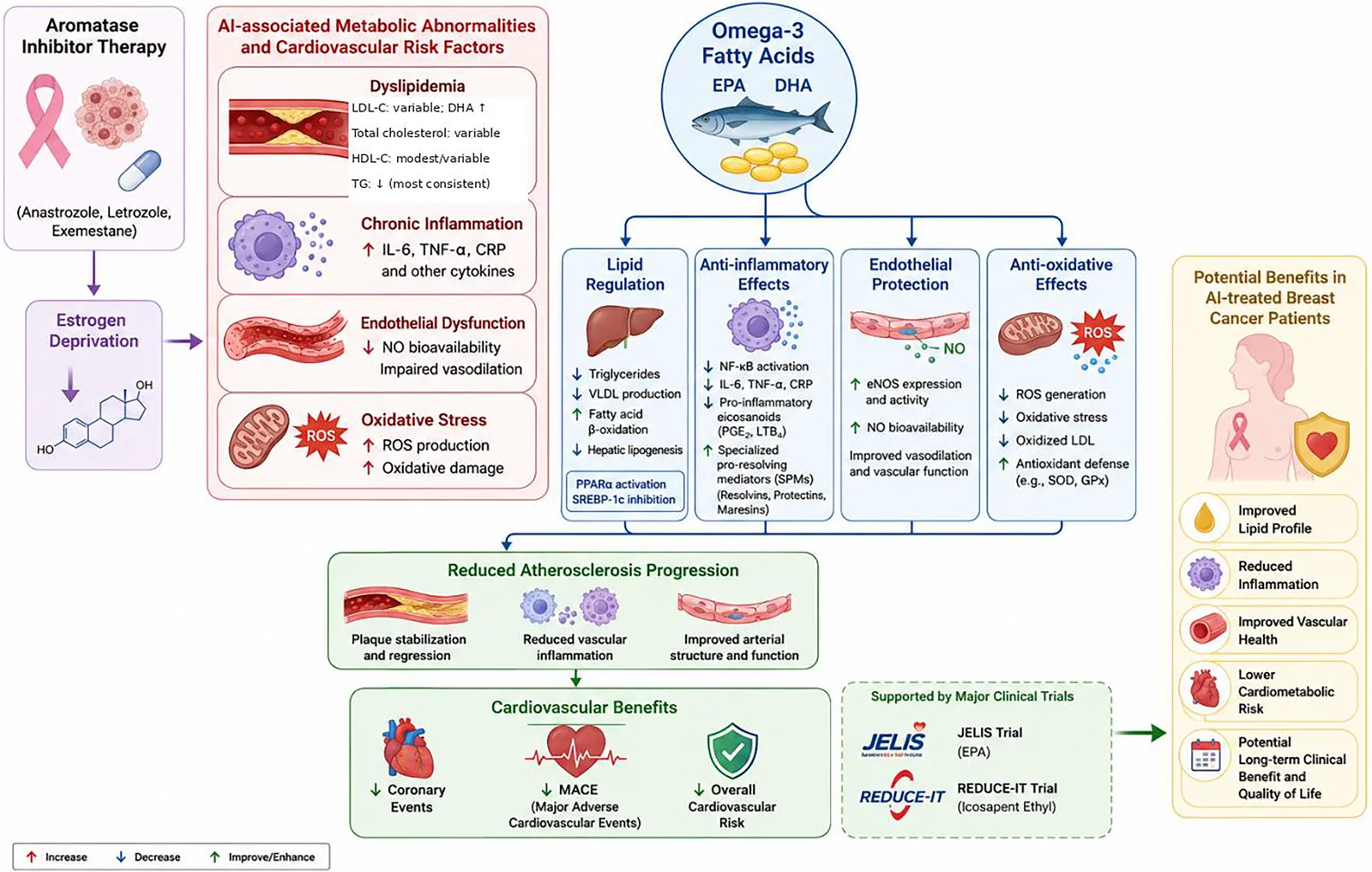
Proposed mechanisms by which omega-3 fatty acids may mitigate aromatase inhibitor-associated metabolic abnormalities and cardiovascular risk.Omega-3 fatty acids may improve lipid metabolism, suppress chronic inflammation, enhance endothelial function, and reduce oxidative stress. These interconnected mechanisms may contribute to the attenuation of aromatase inhibitor (AI)-associated dyslipidemia and cardiovascular risk, ultimately supporting long-term metabolic and cardiovascular health in breast cancer survivors receiving endocrine therapy. AI, aromatase inhibitor; EPA, eicosapentaenoic acid; DHA, docosahexaenoic acid; LDL-C, low-density lipoprotein cholesterol; HDL-C, high-density lipoprotein cholesterol; VLDL, very-low-density lipoprotein; IL-6, interleukin-6; TNF-α, tumor necrosis factor-α; CRP, C-reactive protein; ROS, reactive oxygen species; eNOS, endothelial nitric oxide synthase; SOD, superoxide dismutase; GPx, glutathione peroxidase; MACE, major adverse cardiovascular events.

Nevertheless, mechanistic plausibility should not be equated with demonstrated clinical efficacy. The majority of clinical trials supporting omega-3 therapy were performed in patients with primary or secondary hypertriglyceridemia rather than patients with breast cancer receiving AIs. Moreover, these studies generally evaluated TG reduction rather than AI-associated changes in LDL-C, TC, or HDL-C. This distinction is particularly important because AI-associated dyslipidemia may involve changes in multiple lipid fractions rather than isolated hypertriglyceridemia.

The formulation of omega-3 therapy represents another important source of heterogeneity. EPA and DHA should not be considered metabolically interchangeable. High-dose EPA formulations, particularly icosapent ethyl, have demonstrated cardiovascular outcome benefits in selected high-risk populations (31, 32), whereas combined EPA+DHA formulations may produce different effects on LDL-C and cardiovascular outcomes (27). Therefore, future studies in AI-treated breast cancer populations should evaluate EPA and DHA separately rather than treating all omega-3 preparations as a single therapeutic category.

Dose is another important consideration. Available evidence suggests that clinically meaningful TG lowering generally requires approximately 2–4 g/day of EPA and/or DHA, with the magnitude of response influenced by baseline TG concentrations and formulation (25, 26). However, the optimal dose for AI-associated dyslipidemia has not been established. Importantly, extrapolating the 4 g/day dose used in cardiovascular outcome trials directly to breast cancer survivors would be inappropriate without dedicated safety and efficacy data.

The available evidence also raises important safety considerations. Omega-3 supplementation is generally well tolerated, with gastrointestinal symptoms being the most commonly reported adverse effects (38). However, high-dose omega-3 therapy has been associated with a potential increase in atrial fibrillation risk, and the magnitude of bleeding risk may vary according to formulation and concomitant antithrombotic therapy (39, 40). These considerations are particularly relevant for cancer survivors with multiple cardiovascular comorbidities or concomitant medications.

Overall, current evidence supports omega-3 fatty acids as a biologically plausible adjunctive intervention, particularly for patients with hypertriglyceridemia or residual cardiovascular risk, but it does not establish omega-3 supplementation as a first-line treatment for AI-associated dyslipidemia. Standard lipid management, including lifestyle modification and guideline-directed lipid-lowering therapy when indicated, should remain the foundation of treatment. Future randomized controlled trials specifically enrolling AI-treated patients with breast cancer are required to determine whether omega-3 supplementation improves lipid profiles and whether such changes translate into meaningful cardiovascular or survivorship benefits.

### 5.4 Cardiovascular evidence and its limitations

Cardiovascular outcome evidence provides additional support for the potential clinical relevance of omega-3 fatty acids, particularly purified EPA. In REDUCE-IT, icosapent ethyl (4 g/day) reduced major cardiovascular events by approximately 25% in statin-treated patients with elevated TG and established cardiovascular disease or diabetes plus additional risk factors (31). Similarly, JELIS demonstrated a reduction in major coronary events with EPA added to statin therapy (18). However, cardiovascular benefits have not been consistently demonstrated across all omega-3 formulations, with outcomes influenced by formulation, dose, background therapy, and baseline cardiovascular risk (28, 32).

These findings should therefore not be directly extrapolated to women receiving AIs, who may have different baseline cardiovascular risk profiles. Moreover, cardiovascular benefits cannot be assumed solely from improvements in lipid parameters. Thus, omega-3 fatty acids may represent a promising adjunctive strategy for AI-treated patients with dyslipidemia or elevated cardiovascular risk, but dedicated trials are needed to determine whether lipid improvements translate into meaningful cardiovascular benefits in this population (**Table 3**).

**Table 3:** Clinical studies evaluating omega-3 fatty acid supplementation in patients with breast cancer.

Study	Population	Intervention	Evidence signal	Major limitation
Yee et al.	Breast cancer risk population	EPA+DHA	Tissue incorporation demonstrated	No AI therapy; no lipid endpoint
REDUCE-IT	High-risk CVD	Icosapent ethyl 4 g/day	MACE reduction	Not breast cancer/AI population
Breast cancer systematic review	Breast cancer	Omega-3	Possible metabolic benefit	Heterogeneous endocrine therapy
AI population	AI-treated breast cancer	–	Direct evidence lacking Direct evidence lacking	No dedicated RCT

EPA, eicosapentaenoic acid; DHA, docosahexaenoic acid.

### 5.5 Safety and tolerability of omega-3 fatty acids

Patients with breast cancer receiving long-term aromatase inhibitor therapy may require adjunctive interventions that provide metabolic benefits while maintaining a favorable long-term safety and tolerability profile. Because adjuvant endocrine therapy typically continues for 5–10 years, the potential benefits and risks of supportive metabolic interventions should be carefully considered.

Available evidence indicates that omega-3 fatty acids are generally well tolerated. Recent systematic reviews and meta-analyses of randomized controlled trials have reported no significant increase in serious adverse events with omega-3 fatty acid supplementation, with overall safety profiles comparable to placebo (38).Within the commonly used dose range of 1–4 g/day, discontinuation rates are generally low, and treatment adherence appears to be favorable.

Gastrointestinal symptoms are among the most frequently reported adverse events associated with omega-3 fatty acid supplementation and include fishy aftertaste, reflux, nausea, abdominal discomfort, bloating, diarrhea, and dyspepsia (38). These adverse effects are generally mild and transient, and they rarely require treatment discontinuation. Tolerability may be improved by administering omega-3 fatty acids with meals, dividing the daily dose, or using highly purified pharmaceutical-grade formulations (38).

Potential bleeding risk has been considered because of the modest antiplatelet effects of omega-3 fatty acids. However, a recent meta-analysis involving more than 120,000 participants found no significant increase in overall bleeding risk associated with omega-3 fatty acid use (39). A small increase in bleeding events was observed with high-dose purified EPA formulations, although the absolute increase in risk was limited. Overall, omega-3 fatty acids appear to have a favorable safety profile, although caution may be warranted in patients with active bleeding disorders or severe coagulation abnormalities.

Increasing attention has been directed toward the potential association between high-dose omega-3 fatty acid supplementation and atrial fibrillation. The REDUCE-IT trial was among the major studies to report a modestly higher incidence of atrial fibrillation among patients receiving icosapent ethyl at 4 g/day compared with placebo (31). Subsequent meta-analyses have similarly suggested that high-dose omega-3 fatty acid therapy may be associated with a small increase in atrial fibrillation risk, particularly among individuals with established cardiovascular disease or pre-existing risk factors for arrhythmia (40, 41). However, the absolute increase in risk appears to be relatively small, and the clinical significance of this potential adverse effect may depend on baseline cardiovascular and arrhythmic risk.

Evidence from breast cancer populations has also generally indicated a favorable tolerability profile. Available studies have not demonstrated adverse effects on adherence to endocrine therapy, nor have they identified increased risks of cancer recurrence or other tumor-related outcomes associated with omega-3 fatty acid supplementation. A recent systematic review including patients with breast cancer reported that omega-3 fatty acids were generally well tolerated, with most reported adverse events consisting of mild and reversible gastrointestinal symptoms (37). However, evidence regarding long-term safety remains limited, particularly in patients receiving prolonged AI therapy. Potential effects on lipid metabolism, inflammatory status, and cardiovascular risk may provide additional clinical relevance for breast cancer survivors receiving long-term endocrine therapy, although these potential benefits require further prospective evaluation.

Overall, current evidence supports the generally favorable tolerability of omega-3 fatty acids, while recognizing that high-dose formulations may be associated with small increases in the risks of atrial fibrillation and bleeding in selected populations. However, the absolute incidence and clinical significance of these events may vary according to dose, formulation, baseline cardiovascular risk, and concomitant medications. Therefore, the long-term risk–benefit profile of omega-3 supplementation specifically in AI-treated breast cancer populations remains to be established through dedicated prospective studies.

Omega-3 fatty acids should be considered complementary rather than substitutive to conventional lipid-lowering therapy. Statins remain a cornerstone of lipid management because of their established efficacy in reducing low-density lipoprotein cholesterol (LDL-C) and preventing atherosclerotic cardiovascular disease (42, 43). In contrast, the primary lipid-lowering effect of omega-3 fatty acids is observed on triglycerides (TG), whereas their effects on LDL-C are generally modest and formulation-dependent. Accordingly, statin-based therapy should remain central to the management of AI-associated dyslipidemia characterized predominantly by elevated LDL-C levels. In patients with concomitant hypertriglyceridemia, metabolic syndrome, or persistent residual cardiovascular risk, omega-3 fatty acids may be considered as an adjunctive therapeutic option when clinically appropriate (27, 43).

Current cardiovascular guidelines support the use of prescription-grade omega-3 fatty acids as an adjunctive treatment option in selected patients with hypertriglyceridemia, while recognizing the potential risk of atrial fibrillation (43). Accordingly, individualized cardiovascular risk assessment and appropriate monitoring are important when considering omega-3 supplementation in patients receiving long-term AI therapy. However, whether such an approach translates into meaningful cardiovascular or survivorship benefits in AI-treated breast cancer populations remains uncertain (**Table 4**).

**Table 4:** Comparison of omega-3 fatty acids and statins in the management of dyslipidemia and cardiovascular risk.

Parameter	Omega-3 fatty acids	Statins
Primary lipid target	Triglycerides (TG)	Low-density lipoprotein cholesterol (LDL-C)
Effect on LDL-C	Minimal to modest	Marked reduction
Effect on TG	Moderate to substantial reduction	Mild to moderate reduction
Anti-inflammatory properties	Present	Present
Cardiovascular outcome benefit	Demonstrated in selected high-risk populations, particularly with high-dose EPA formulations	Well established across a broad range of cardiovascular risk populations
Tolerability	Generally well tolerated; gastrointestinal symptoms most common	Generally well tolerated; muscle-related symptoms and liver enzyme elevations may occur
Major safety concern	Potential increase in atrial fibrillation risk at high doses	Myopathy, liver enzyme elevation, and rare intolerance
Clinical role in AI-associated dyslipidemia	Adjunctive therapy, particularly in patients with hypertriglyceridemia or residual cardiovascular risk	First-line pharmacological therapy, especially in patients with elevated LDL-C
Overall clinical positioning	Complementary metabolic intervention	Foundation of lipid-lowering therapy

AI, aromatase inhibitor; EPA, eicosapentaenoic acid; LDL-C, low-density lipoprotein cholesterol; TG, triglyceride.

## 6 Future perspectives and research directions

### 6.1 Lack of direct clinical evidence for AI-associated dyslipidemia

Although substantial evidence supports omega-3 fatty acids for the management of hypertriglyceridemia and cardiovascular risk in selected populations, direct clinical evidence regarding their efficacy in aromatase inhibitor (AI)-associated dyslipidemia remains limited. Current evidence is derived primarily from studies in general hyperlipidemic populations, breast cancer populations, and mechanistic investigations. To date, well-designed randomized controlled trials specifically evaluating omega-3 supplementation in patients with AI-associated dyslipidemia are lacking (**Table 5**).

**Table 5:** Current evidence and knowledge gaps regarding omega-3 fatty acids in AI-associated dyslipidemia.

Evidence domain	Current findings	Overview of omega-3 fatty acid doses	Supporting references	Limitations
Biological plausibility	AI-induced estrogen deprivation contributes to dyslipidemia and cardiovascular risk; omega-3 fatty acids exert lipid-modifying and cardioprotective effects	Experimental and clinical studies have evaluated a broad range of omega-3 doses, with metabolic effects generally more evident at higher doses	(7–9, 25)	Evidence is largely mechanistic and indirect
Lipid-lowering efficacy	Omega-3 fatty acids consistently reduce TG levels, with more modest and variable effects on LDL-C, TC, and HDL-C	Approximately 2–4 g/day of EPA and/or DHA is commonly used for clinically meaningful TG lowering; prescription EPA has been studied at 4 g/day	(25, 31, 33–35)	Most studies were not conducted in breast cancer populations
Breast cancer population evidence	Omega-3 supplementation increases breast tissue omega-3 incorporation and may improve metabolic and inflammatory profiles	Breast cancer studies have used a broad range, including 0.84–7.56 g/day EPA+DHA in Yee et al. and ~1.81 g/day n-3 fatty acids in Paixão et al.	(10, 12, 37)	Lipid outcomes are rarely primary study endpoints
Evidence specific to AI-treated patients	No large randomized controlled trial has specifically evaluated omega-3 fatty acids for AI-associated dyslipidemia	No evidence-based AI-specific dose has been established.	—	Direct clinical evidence remains lacking
Cardiovascular risk reduction	High-dose EPA significantly reduces major adverse cardiovascular events in high-risk populations	Icosapent ethyl 4 g/day in REDUCE-IT; EPA 1.8 g/day in JELIS.	(31–33)	Applicability to AI-treated patients with breast cancer remains uncertain
Safety and tolerability	Generally well tolerated; gastrointestinal symptoms are the most common adverse events, while potential risks of atrial fibrillation and bleeding require further evaluation	Higher-dose regimens, particularly 4 g/day, require consideration of long-term safety and tolerability	(38–40)	Long-term safety data in AI-treated populations are limited
Future research priorities	Well-designed RCTs focusing on lipid profiles, inflammatory biomarkers, cardiovascular outcomes, and optimal EPA/DHA formulations in AI-treated patients are needed	Future trials should compare different doses and formulations, including EPA-only 、DHA-only and EPA+DHA preparations	—	Major evidence gap

AI, aromatase inhibitor; EPA, eicosapentaenoic acid; DHA, docosahexaenoic acid; LDL-C, low-density lipoprotein cholesterol; TG, triglyceride.

AI-associated dyslipidemia is also characterized by substantial biological and methodological heterogeneity. Estrogen deprivation caused by aromatase inhibition may alter hepatic lipoprotein metabolism, endothelial function, inflammatory pathways, and cardiovascular homeostasis (7, 8). However, the magnitude and direction of lipid changes vary across individual AIs, treatment duration, baseline metabolic status, and prior endocrine therapy (4, 5, 8). A 2024 systematic review and meta-analysis of 15 studies involving 1,708 patients similarly found heterogeneous lipid responses according to AI type and treatment duration, underscoring that AI-associated dyslipidemia should not be regarded as a uniform metabolic phenotype (8).

An important methodological consideration in interpreting these findings is the choice of comparator. Much of the evidence suggesting an unfavorable lipid profile with AIs has been generated from comparisons with tamoxifen rather than with a metabolically neutral comparator. This distinction is important because tamoxifen itself has lipid-modifying effects, particularly reductions in total cholesterol and LDL-C, and therefore the apparent difference between an AI and tamoxifen may reflect both estrogen deprivation following aromatase inhibition and the loss of tamoxifen-associated lipid effects (15). In the BIG 1–98 trial, for example, low-grade hypercholesterolemia was reported more frequently with letrozole than with tamoxifen, although such comparisons cannot by themselves determine how much of the observed difference is attributable to letrozole versus the lipid-lowering effect of tamoxifen (4).

Evidence from longitudinal studies further supports the need to distinguish direct AI effects from tamoxifen withdrawal. In 190 postmenopausal women who discontinued 5–7 years of tamoxifen therapy without subsequent treatment, total cholesterol and LDL-C increased significantly within 6 months, whereas HDL-C did not change significantly, suggesting that the favorable lipid effects of tamoxifen can diminish relatively rapidly after treatment cessation (44). Similarly, a prospective study of 186 postmenopausal women receiving AIs found that increases in cholesterol and inflammatory markers occurred predominantly among patients previously treated with tamoxifen, while no significant lipid changes were observed in patients receiving AIs as first-line endocrine therapy; the authors therefore proposed that tamoxifen withdrawal might account for part of the observed changes (45). These findings are hypothesis-generating rather than definitive evidence of causality, because treatment sequence, baseline metabolic characteristics, and other clinical factors may differ between patients who switch from tamoxifen and those who initiate AIs directly. Consistent with this uncertainty, a large nationwide Danish cohort of 10,461 postmenopausal women published in 2025 found no evidence that AI treatment adversely affected LDL-C or other major lipid parameters compared with women not receiving AIs (46).

This attribution problem has important implications for future omega-3 research. If the lipid phenotype observed after switching from tamoxifen to an AI partly reflects withdrawal of tamoxifen-associated lipid protection, the expected benefit of omega-3 supplementation may differ from that in patients who initiate an AI as first-line endocrine therapy. Future prospective studies should therefore incorporate appropriate non-tamoxifen comparators, standardized fasting lipid measurements, baseline and serial assessment of lipid profiles, detailed documentation of prior tamoxifen exposure, and adjustment for concomitant lipid-lowering therapy and baseline cardiovascular risk. Ideally, studies should distinguish patients initiating AIs as first-line endocrine therapy from those switching from tamoxifen to an AI and should evaluate lipid trajectories before and after the endocrine treatment transition. Such designs would help disentangle metabolic changes attributable to estrogen deprivation from those associated with tamoxifen withdrawal and provide a more accurate estimate of the lipid effects of individual AIs.

More attention has recently shifted toward maintaining metabolic health in breast cancer survivorship. Cardiovascular disease remains a major non-cancer threat to long-term outcomes among breast cancer survivors receiving prolonged endocrine therapy, and cardio-oncology and survivorship guidelines recommend ongoing assessment and management of dyslipidemia and cardiovascular risk factors (42, 43, 47). Future research should therefore prioritize well-designed prospective randomized controlled trials that specifically enroll patients with AI-associated dyslipidemia. Primary endpoints should include changes in LDL-C, TG, and non-HDL-C, while secondary outcomes may include inflammatory biomarkers, measures of insulin resistance, endothelial function, quality of life, treatment adherence, and cardiovascular risk scores. Because different AIs may have different lipid effects (4, 5, 8), future studies should also determine whether the metabolic effects of omega-3 fatty acids vary according to the specific AI agent used.

Adjuvant endocrine therapy often extends beyond 5 years, making long-term management of treatment-related metabolic complications increasingly important. Large multicenter studies with extended follow-up are needed to determine not only the lipid-modifying efficacy of omega-3 fatty acids but also their effects on cardiovascular outcomes, treatment adherence, and long-term survivorship. Together, these studies would help define the clinical role of omega-3 fatty acids in AI-associated dyslipidemia and provide a stronger evidence base for individualized metabolic and cardiovascular management.

### 6.2 Optimisation strategies for omega-3 fatty acid dosage and formulation

Substantial evidence supports the use of omega-3 fatty acids for the management of hypertriglyceridemia and suggests potential cardiovascular benefits in selected populations. However, the optimal dose, formulation, and treatment strategy for AI-associated dyslipidemia have not been established. Existing studies have used different EPA-to-DHA ratios, daily doses, treatment durations, and study populations, which limits direct comparison across studies and complicates the extrapolation of existing evidence to patients receiving AI therapy.

Different omega-3 formulations may have distinct metabolic and cardiovascular effects. Meta-analyses have suggested that EPA-only formulations may provide greater cardiovascular benefit than combined EPA+DHA preparations in certain high-risk populations (32, 33). The REDUCE-IT trial demonstrated that high-purity EPA (icosapent ethyl, 4 g/day) reduced major adverse cardiovascular events in statin-treated patients with elevated cardiovascular risk (31). Future studies of AI-associated dyslipidemia should evaluate both the overall efficacy of omega-3 fatty acids and potential differences between EPA-only and EPA+DHA formulations. Whether EPA monotherapy provides greater metabolic or cardiovascular benefit than combined EPA+DHA formulations in patients receiving AI therapy remains uncertain.

Dose optimization represents another important research priority. Available evidence indicates a dose-dependent reduction in triglyceride concentrations with omega-3 supplementation (25).However, higher doses may be associated with an increased risk of adverse events, particularly atrial fibrillation (40, 41). Because adjuvant endocrine therapy may continue for 5–10 years, the long-term benefit–risk profile of different omega-3 doses requires further evaluation in patients receiving AI therapy. Head-to-head trials comparing different dose regimens, such as 1 g/day, 2 g/day, and 4 g/day, are needed to determine the minimum effective dose and the most appropriate benefit–risk profile for patients with AI-associated dyslipidemia.

Formulation and dose should also be considered within the broader context of comprehensive metabolic management. Current guidelines recommend tailoring lipid-lowering treatment according to individual cardiovascular risk, lipid profiles, and comorbidities (48). In patients with persistent hypertriglyceridemia, prescription omega-3 fatty acids may be considered as an adjunct to statin therapy in appropriate clinical settings (26, 48). Patients receiving AI therapy may also have obesity, metabolic syndrome, insulin resistance, or other cardiometabolic risk factors. Future studies should therefore evaluate the potential effects of omega-3 fatty acids when combined with statins, lifestyle interventions, weight-management strategies, and other cardiometabolic approaches.

Determining the appropriate omega-3 formulation, dose, and combination with other interventions is important for defining its clinical role in AI-associated dyslipidemia. Future high-quality randomized trials and real-world studies should evaluate formulation-specific effects, dose–response relationships, long-term cardiovascular outcomes, and patient-centered outcomes. These studies may provide the evidence needed to develop standardized and individualized strategies for the management of AI-associated dyslipidemia.

### 6.3 Precision nutrition and individualised intervention strategies

With the continued development of precision medicine and precision nutrition, the potential application of omega-3 fatty acids for AI-associated dyslipidemia warrants consideration within a more individualized therapeutic framework rather than a uniform treatment approach. Breast cancer survivors receiving long-term endocrine therapy represent a heterogeneous population with substantial variation in age, metabolic status, cardiovascular risk, treatment regimens, and genetic background. Consequently, responses to omega-3 fatty acid supplementation may vary considerably among individuals. Identifying determinants of treatment response and developing personalized intervention strategies therefore represent important areas for future research.

Baseline metabolic status may influence the response to omega-3 fatty acid supplementation. Individuals with hypertriglyceridemia generally exhibit greater TG-lowering effects, whereas those with normal baseline lipid levels may experience more modest changes in lipid parameters (25, 32). Whether the effects of omega-3 fatty acids differ according to the predominant lipid phenotype in AI-treated patients, including hypertriglyceridemia, elevated LDL-C, or mixed dyslipidemia, remains unclear. Future studies should evaluate treatment responses across these lipid phenotypes to identify patient subgroups that may derive greater benefit from omega-3 supplementation.

Genetic factors involved in lipid metabolism and inflammatory pathways may also influence individual responses to omega-3 fatty acids (49). Interindividual differences in inflammatory signaling, lipoprotein metabolism, and fatty acid oxidation capacity may further contribute to variability in treatment response. Nutrigenomic approaches may therefore help identify patient subgroups with distinct metabolic responses to omega-3 supplementation and inform the development of more individualized nutritional interventions (49).

Biomarker-guided nutritional interventions may further refine treatment stratification beyond genetic information alone. The Omega-3 Index, which reflects the proportion of EPA and DHA in erythrocyte membranes, has been proposed as a biomarker of longer-term omega-3 status and has been associated with cardiovascular risk and clinical outcomes (50). Integrating the Omega-3 Index with metabolic profiling, genetic information, and validated risk prediction models may facilitate the development of more individualized nutritional intervention strategies and help identify patients who are more likely to benefit from omega-3 supplementation (51).

Future research should therefore move beyond a conventional lipid-centered approach toward a precision nutrition framework for omega-3 supplementation. Individualized intervention strategies incorporating clinical characteristics, metabolic phenotypes, genetic information, and biomarker profiles may help optimize the use of omega-3 fatty acids in breast cancer survivors receiving long-term endocrine therapy. However, the clinical utility of such approaches remains to be established through prospective studies incorporating validated biomarkers, predefined metabolic phenotypes, and clinically relevant outcomes.

### 6.4 Proposed research framework for future clinical trials

Future studies on aromatase inhibitor (AI)-associated dyslipidemia should extend beyond lipid profile improvement to evaluate broader metabolic effects and long-term clinical outcomes. Conventional lipid parameters, including low-density lipoprotein cholesterol (LDL-C), triglycerides (TG), high-density lipoprotein cholesterol (HDL-C), and non-high-density lipoprotein cholesterol (non-HDL-C), may not fully capture the metabolic and vascular effects of omega-3 fatty acid supplementation. Future clinical trials should therefore consider incorporating biomarkers reflecting inflammation, insulin resistance, and vascular function, such as high-sensitivity C-reactive protein (hs-CRP), interleukin-6 (IL-6), the homeostasis model assessment of insulin resistance (HOMA-IR), flow-mediated dilation (FMD), and carotid intima–media thickness (cIMT). Collectively, these measures may provide a more comprehensive assessment of the potential effects of omega-3 fatty acids on AI-associated metabolic abnormalities (32, 47, 48).

Because adjuvant AI therapy may continue for 5–10 years, long-term follow-up is needed to determine whether improvements in lipid profiles translate into clinically meaningful outcomes. Future studies should systematically evaluate major adverse cardiovascular events (MACE), treatment adherence, quality of life, breast cancer recurrence, and overall survival. In addition, given the differential effects of anastrozole, letrozole, and exemestane on lipid metabolism (4, 5, 8), randomized controlled trials should consider stratified analyses according to the specific AI agent to determine whether the metabolic effects of omega-3 fatty acid supplementation vary across treatment regimens.

With the continued development of precision medicine and precision nutrition, integration of the Omega-3 Index, polymorphisms in genes involved in fatty acid metabolism (e.g., the FADS gene cluster), and emerging multi-omics technologies may facilitate the development of individualized intervention models based on patient-specific risk stratification (49, 52). Such approaches may help identify patient subgroups that are more likely to benefit from omega-3 fatty acid supplementation and inform the development of personalized nutritional strategies for the management of AI-associated dyslipidemia.

Standardized outcome measures, long-term follow-up frameworks, and individualized risk assessment strategies will be important components of future clinical research. Establishing these elements may facilitate the generation of higher-quality clinical evidence regarding the role of omega-3 fatty acids in the metabolic management of breast cancer survivors. Whether omega-3 supplementation can ultimately improve cardiovascular health and long-term clinical outcomes in patients receiving AI therapy will require confirmation in well-designed prospective studies.

**Figure 4** summarizes potential strategies for addressing current evidence gaps and facilitating the translation of omega-3 fatty acid research into clinical practice. Key considerations for future trial design include comprehensive baseline metabolic assessment, cardiovascular risk stratification, head-to-head comparisons of different omega-3 formulations and dosages, long-term follow-up, and precision nutrition–based intervention strategies.

**Figure 4:**
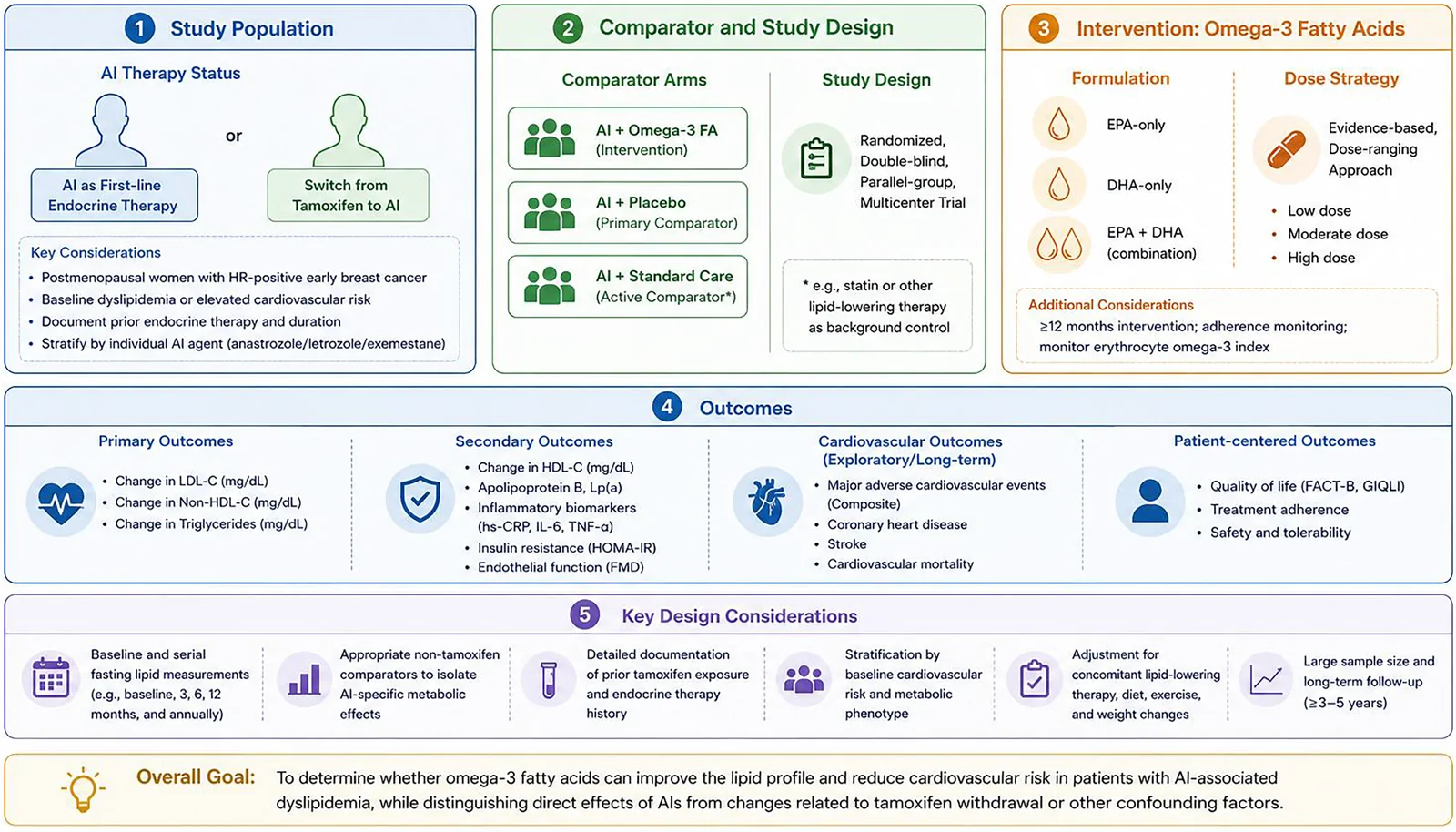
Proposed framework for future clinical trials in AI-associated dyslipidemia. Key design elements to address current knowledge gaps and advance precision management. The framework outlines (1) study population criteria based on AI therapy status, (2) comparator arms and randomized double-blind parallel-group multicenter trial design, (3) omega-3 fatty acid intervention strategies including formulation and dose-ranging approaches, (4) hierarchical outcome measures spanning primary lipid endpoints, secondary metabolic and inflammatory biomarkers, exploratory cardiovascular events, and patient-centered outcomes, and (5) key methodological considerations to ensure robust trial execution and interpretation. AI, aromatase inhibitor; DHA, docosahexaenoic acid; EPA, eicosapentaenoic acid; FA, fatty acid; FACT-B, Functional Assessment of Cancer Therapy–Breast; FMD, flow-mediated dilation; GIQLI, Gastrointestinal Quality of Life Index; HDL-C, high-density lipoprotein cholesterol; HOMA-IR, homeostatic model assessment of insulin resistance; hs-CRP, high-sensitivity C-reactive protein; IL-6, interleukin-6; LDL-C, low-density lipoprotein cholesterol; Lp(a), lipoprotein(a); Non-HDL-C, non-high-density lipoprotein cholesterol; TNF-α, tumor necrosis factor-α.

## 7 Conclusion

Aromatase inhibitors (AIs) have substantially improved outcomes among patients with hormone receptor-positive breast cancer. However, AI-associated metabolic disturbances and their potential cardiovascular consequences have increasingly gained attention in long-term survivorship care. Persistent estrogen deprivation during AI therapy may be associated with adverse alterations in lipid metabolism, including increases in low-density lipoprotein cholesterol (LDL-C) and total cholesterol (TC), accompanied by endothelial dysfunction, oxidative stress, and chronic low-grade inflammation. Collectively, these metabolic and vascular alterations may contribute to the progression of atherosclerosis and an increased long-term cardiovascular risk among breast cancer survivors.

Omega-3 polyunsaturated fatty acids, particularly eicosapentaenoic acid (EPA) and docosahexaenoic acid (DHA), have well-established triglyceride-lowering effects. They may also exert broader cardiovascular effects through modulation of lipid metabolism, inflammatory pathways, and vascular function. Evidence from mechanistic studies, randomized controlled trials, and large cardiovascular outcome studies has demonstrated the potential of omega-3 fatty acids to improve selected metabolic abnormalities and reduce cardiovascular risk in specific clinical populations (25, 31, 32). In patients with breast cancer receiving long-term AI therapy, omega-3 fatty acids may therefore represent a potential adjunctive intervention alongside lifestyle modification and conventional lipid-lowering therapy. However, direct clinical evidence supporting their efficacy specifically for AI-associated dyslipidemia remains limited, and their role in this population requires further prospective investigation.

Overall, omega-3 fatty acids have a strong biological rationale and established triglyceride-lowering efficacy in broader clinical populations, but their efficacy in AI-associated dyslipidemia remains unproven. The current evidence is constrained by indirectness, heterogeneity in AI exposure and omega-3 formulations, limited AI-specific clinical data, and uncertainty regarding the independent contribution of AI therapy versus tamoxifen withdrawal to observed lipid changes. Omega-3 fatty acids should therefore be considered a potential adjunctive strategy rather than an established treatment for AI-associated dyslipidemia. Well-designed, adequately powered prospective trials with appropriate comparators, standardized lipid phenotyping, formulation- and dose-specific interventions, and long-term cardiovascular and oncological follow-up are required before their clinical role can be defined.
